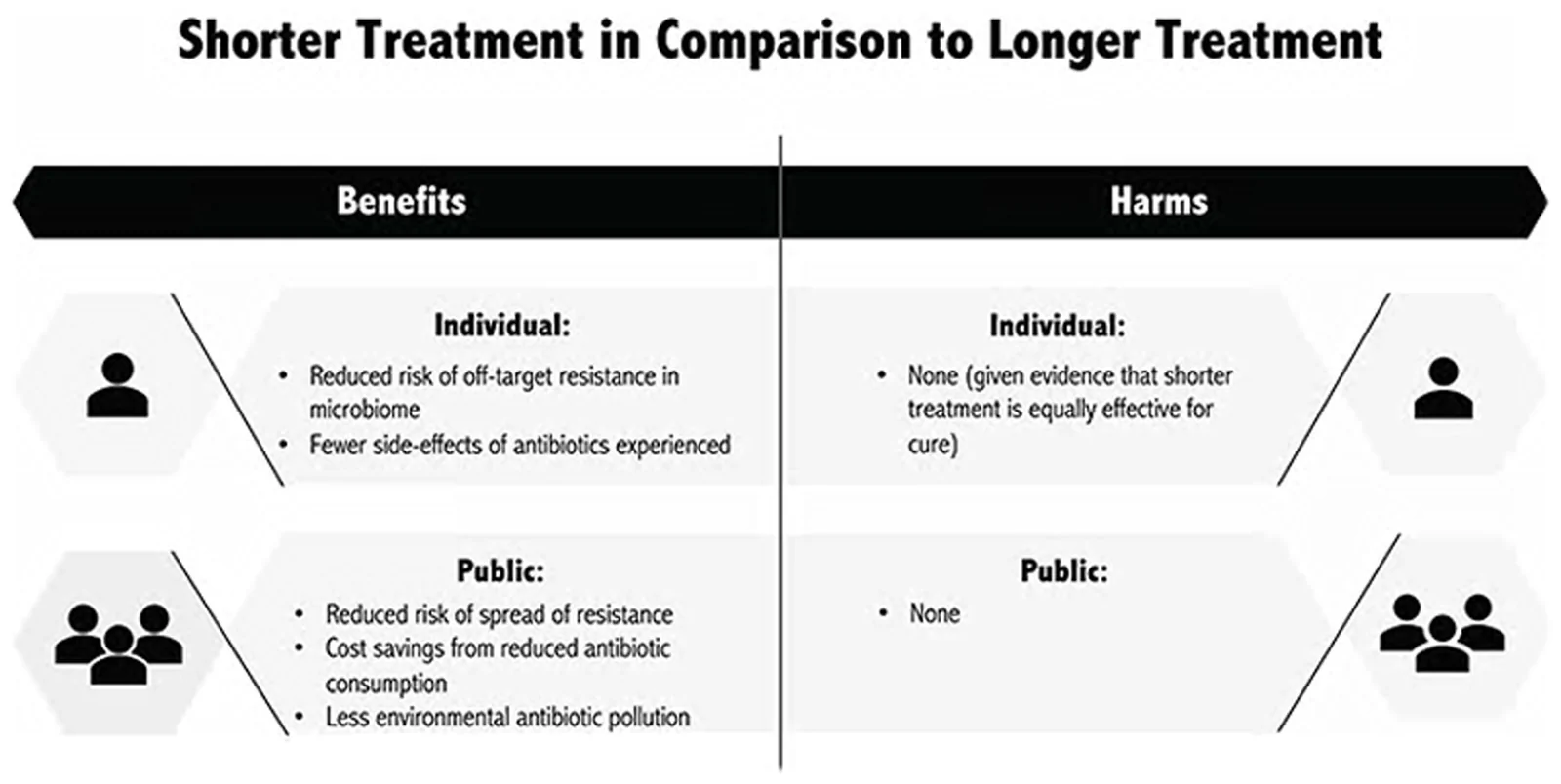# Ethics of Antibiotic Course Duration: Shorter is Better

**DOI:** 10.1080/15265161.2026.2632006

**Published:** 2026-03-06

**Authors:** Tess Johnson, George S Heriot, Euzebiusz Jamrozik

**Affiliations:** 1 Ethox Centre, Oxford Population Health, University of Oxford, Oxford, UK; 2 Pandemic Sciences Institute, Nuffield Department of Medicine, University of Oxford, Oxford, UK; 3 Department of Infectious Diseases University of Melbourne, Peter Doherty Institute for Infection and Immunity, Melbourne, Australia; 4 Monash Bioethics Centre, Monash University, Melbourne, Australia

**Keywords:** medical ethics, drug resistance, antibiotics, prescription duration, evidence-based medicine

## Abstract

Antibiotic treatment course duration has for decades been dictated by two questionable ideas: first, that longer courses are more effective at curing bacterial infections; second, that longer courses are less likely to lead to drug resistance. Recently, the “shorter is better” movement has challenged the received wisdom, showing shorter-duration antibiotic courses provide similar cure rates, fewer antibiotic-related harms, and possibly less contribution to of antibiotic resistance for common infections. Yet, physicians typically still prescribe longer courses of antibiotics than clinical guidelines recommend. This is ethically unacceptable. In this paper, we argue that prescribing physicians are ethically obligated to prescribe shorter antibiotic courses, given that prescribing physicians bear duties both toward their patients and to protect public health. We rebut objections to our argument and furthermore argue for an ethical obligation of physician-researchers to conduct further trials comparing the shortest current evidence-based course of antibiotics with courses that are even shorter.

## Introduction

Antibiotic resistance (ABR) is a significant global challenge, posing current and future harm to populations from the spread of bacterial infections that do not respond to antibiotics. ABR already causes over 1.27 million deaths per year and may cause up to 10 million deaths per year by 2050 (O’Neill 2016). Stewardship of effective antibiotics is essential. Here we focus on medical uses of antibiotics in humans. To mitigate the expected increase in mortality, those people who use or prescribe antibiotics must protect the existing stock of effective antibiotics from becoming ineffective. It is widely assumed (by physicians, bioethicists, and laypeople alike) that failure to “finish the course” of antibiotics or the prescription of a course that is too short to clear the target infection is a major contributor to the prevalence of resistant bacteria (Anomaly 2010; Borek et al. 2023; Llewelyn et al. 2017). Where such courses are longer than the minimum duration required to clear a patient’s infection, completing the course may result in too long a treatment duration. What’s more, this antibiotic stewardship educational messaging is not supported by evidence. The data do not support the assumption that longer courses are less likely to contribute to ABR or that “finishing the course” is the best approach to antibiotic stewardship (Rice 2008). As long as prescribed courses are too long, in fact, advice to “finish the course” may cause harm (Mo, Tan and Cooper 2025). The risks of our actions undermining antibiotic effectiveness have been recognized since Alexander Fleming, who won the Nobel Prize for the discovery of the antibiotic properties of penicillin, claimed that

the thoughtless person playing with penicillin treatment is morally responsible for the death of the man who succumbs to infection with the resistant organism. (New York Times 1945, 21)

It is unclear whether Fleming was aware of several studies conducted in the two years before he accepted the Nobel showing effective treatment of pneumococcal pneumonia with shorter courses of penicillin (Rice 2008). Certainly today, that knowledge appears to be lost: the common view is that patients should complete long courses of antibiotics (Pandolfo et al. 2022)—and patients who fail to do so are negatively moralized (Kraaijeveld and Jamrozik 2022). Recent studies of physician practices have highlighted a tendency among physicians to prescribe longer courses than the length recommended in current guidelines (let alone the length supported by recent evidence from clinical trials, which is often shorter even than is recommended by guidelines). For respiratory tract indications, 70% or more of antibiotic treatment courses prescribed in the USA exceed guideline recommendations in terms of treatment duration (Yi et al. 2018). Even worse, in the UK, the figure is around 80% (Pouwels et al. 2019). The difference this overprescription makes to overall antibiotic consumption is huge: the overprescription in consultations observed in a UK-based study resulted in 1.3 million additional days of antibiotic prescriptions beyond the length recommended by guidelines (Pouwels et al. 2019). In addition to these concerns of unnecessary antibiotic prescribing in terms of contributing to ABR, evidence indicates that most courses of antibiotics are far too long compared to what is required to cure the patient of the target infection, as reflected in the growing evidence-based movement in infectious disease medicine arguing that, when it comes to antibiotic course duration, “shorter is better” (Spellberg 2016).

At this point, the physician may feel confused. Common prescribing practice pulls the physician in one direction, yet both clinical guidelines and recent evidence on shorter course durations pulls them in the other; consideration of adverse effects of antibiotic treatment pull the physician in one direction, yet the worry of failing to stabilize the patient, prevent hospital admission, or clear the infection pull them in the other. Considering this spectrum of evidence, guidance and practice, the physician appears to have the following plausible prescription options:

1. Shorter than guidelines recommend and than colleagues prescribe (but a length supported by clinical evidence);
2. Within the range that guidelines recommend, but shorter than the antibiotic treatment duration their colleagues tend to prescribe;
3. Longer than guidelines recommend, but the same antibiotic treatment duration as their colleagues tend to prescribe.

For our purposes here, we consider ‘shorter courses’ to constitute physician prescription options 1 and 2, and we consider ‘longer courses’ to constitute option 3. These prescribing decisions are ethical in nature, as they concern physicians’ obligations given their duty of care toward their patients and their duties as antibiotic stewards. If the physician does consider certain often-neglected adverse effects of antibiotics for patients, they may perceive there to be a tension in interests that is common public health ethics: the tension between promoting an individual’s good and promoting the collective good. If the physician prescribes a shorter course, they may see themselves as exposing the patient to fewer adverse effects from antibiotics, but at a risk of contributing to ABR from not prescribing a long enough course. If the physician prescribes a longer course, they may see themselves as fulfilling their obligations as an antibiotic steward, but perhaps with some risk of overtreating their patient. Many physicians will recognize no such tension to begin with, simply opting for a “better safe than sorry” approach and prescribing an antibiotic course duration that is longer than that recommended by guidelines, but aligns with common practice. Such an approach may be misguided, insofar as it fails to recognize the risks and harms associated with antibiotic treatment, as we explore further below.

In this paper, we argue that for most cases of common uncomplicated infections such as pneumonia (which constitute the vast majority of clinical antibiotic uses (Pouwels et al. 2019)), physicians face no ethical question about what they should do—rather, they have an ethical obligation to prescribe shorter courses than the current long courses they prescribe (aligning with prescribing option 3), down to the minimum length that clinical guidelines recommend (taking prescribing option 2). We hold that in future, where supported by RCT evidence, physicians will arguably have an ethical obligation to alter their prescription durations down to a shorter duration even than clinical guidelines recommend (taking prescribing option 1). However, this claim depends on whether future guidelines are better updated to align with RCT evidence, on the quality of emerging research evidence, and may be limited to less serious conditions.

In making our argument, we first briefly present what prescribing physicians may perceive to be an ethical question they face when treating a patient with pneumococcal community-acquired pneumonia (CAP). Second, we challenge this perception of there being an ethical question. To do so, we outline evidence that longer courses (5-14 days, wherein physicians opt for prescription option 3) typically provide no additional benefit in terms of curing the patient and involve greater risks than shorter treatment courses (3-5 days, according to guidelines, prescription option 2) (Spellberg, n.d.). We argue that physicians’ ethical obligations toward their patients and for protecting public health both direct them toward prescribing shorter antibiotic courses. Third, we respond to potential objections that might be raised in support of common practice, in a few key areas (i) individual-level practical considerations physicians face; (ii) systemic factors constraining physicians’ plausible range of prescription choices; (iii) inevitable behavioral and cognitive influences. In the final section, we highlight a real ethical question: whether clinical researchers should be conducting trials comparing short antibiotic course durations to even shorter ones. We hold that in the presence of clinical equipoise, further research should be conducted to determine if even shorter antibiotic courses are also non-inferior in curing the patient, and therefore superior overall, considering the effects of antibiotic exposure on patient health and likely contributions to ABR. If such future research is conducted, future clinicians may also have an ethical obligation to prescribe these even shorter courses, even if future guidelines fail to reflect the available evidence. We conclude by exploring the implications of our argument for a range of infectious diseases.

### The Ethical Question of How To Prescribe

As we will explore below, while it is true that exposure of infection-causing bacteria in the human body to antibiotics results in the selection and development of resistant bacteria, resistance often develops from “off-target” effects on the human microbiome rather than in the “target” bacteria causing an episode of clinical infection (See [Figure 1]). Each time a patient takes a dose of an antibiotic, the number and variety of bacteria in their microbiome that are exposed to the antibiotic far outnumber the relatively small numbers of bacteria of involved in what is usually a monomicrobial infection (i.e., caused by one pathogenic species). Shorter courses may result in fewer resistant bacteria in the microbiome (although the evidence-base relating shorter courses to reduced resistance is still somewhat limited (Mo, Tan and Cooper 2025)) and, as research has increasingly demonstrated, shorter courses do not result in lower cure rates for many common infections compared to longer courses—therefore allaying fears that a patient might develop resistant pneumococcal pneumonia (due to insufficiently long treatment duration) and transmit these bacteria to others or experience an escalation in their condition. Patients failing to finish a course or physicians prescribing courses that are too short to cure a target infection (e.g., pneumococcal pneumonia) are therefore not major contributors to the prevalence of antibiotic resistance. Yet, we reiterate that many physicians seem to feel that they are doing the ethically right thing in prescribing longer courses, in accordance with tradition or standard practice in their setting. Prescribers may feel that there is an ethical question surrounding whether to prescribe a shorter or a longer course. This may be based partly on the neglect of off-target effects of antibiotics in the biomedical and ethics literatures that ought to provide support for clinical decision-making (Jamrozik and Heriot 2022).

The issues we wish to discuss here might be realized in a case like this:

A patient presents to their physician with mild to moderate symptoms indicating community-acquired pneumonia (CAP), presumed to be of a bacterial cause, most likely pneumococcus. The physician feels she faces an ethical question regarding what length of antibiotic treatment to prescribe: whilst her colleagues usually prescribe 10 days, and she considers this standard practice, she is aware that recently updated clinical guidance recommends shorter courses.

We have chosen this case example for several reasons. First, there is strong evidence in favor of shorter antibiotic course durations for treating CAP, for both amoxicillin and levofloxin, with 14+ trials conducted and showing non-inferiority of antibiotic treatment durations of 3-5 days compared to treatment durations of 5-14 days (Spellberg, n.d.). Second, this kind of case might be regularly seen by physicians, and with the number of people prescribed antibiotics for CAP, this is a disease for which even small decreases in antibiotic duration across many patients might significantly impact overall population consumption of antibiotics. The greater the difference between typical course length prescribed by physicians, and the shortest evidence-based course length, the stronger our argument is that physicians have an ethical obligation to prescribe shorter treatment duration. Such arguments may be even stronger where antibiotics have more adverse effects or more significant effects on the microbiome than amoxicillin.

There is no question about what the physician in the above scenario should do if she bases her practice simply on clinical guidance. In the US, the Infectious Diseases Society of America (IDSA) sets Practice Guidelines (IDSA n.d.) that are based on expert reviews of available evidence. Implementation also depends on hospital-level guidance, and evidence on drug susceptibilities in the region. Prescribing patterns reveal discrepancies between standards set by the IDSA and hospital-level guidance and prescribing, with greater discrepancies for more severe infections (Rost et al. 2020). In the UK, the National Institute for Health Care Excellence (NICE) sets guidelines (NICE 2019). For treating CAP, NICE sets out a prescribing strategy for those assessed to have mild to moderate pneumonia, recommending that physicians offer antibiotics for adults, young people, and children with CAP within 4 hours of diagnosis (NICE 2019). This immediately excludes two treatment options for the physician—non-prescription and delayed prescription. The first-line treatment for adults with low-severity CAP is 5 days of regular doses of oral amoxicillin. The guidelines further state that physicians should “stop antibiotic treatment after 5 days unless […] a longer course is needed or the person is not clinically stable” (NICE 2019). It seems clear from the guidelines how the physician should act—they should offer a short prescription of (at most) 5 days.

Whether the physician faces an ethical question depends in part on whether they should put stock in current guidance (prescribing option 2), or rather base their prescription decision on their prior teaching, standard practice, evidence in the medical literature, what they believe their duty of care toward their patient or their obligations to protect public health require, or other factors influencing clinical reasoning (likely resulting in their taking prescribing option 3). In future, we might think that if guidelines continue to fail to align with available evidence supporting shorter courses, they should perhaps put stock in the evidence even over guidelines (taking prescribing option 1). Indeed, many antibiotic guidelines regarding course duration are not based on high quality evidence (Rice 2008; Venus and Jamrozik 2020). In that case, does the physician face an ethical question about how to treat the patient?

Our answer is still no. As we will discuss, evidence shows that short antibiotic course durations are equally effective at treating the target infection in most cases. What’s more, shorter courses expose the patient to fewer risks of adverse events. Finally, shorter courses do not lead to increased risk of resistance. Both physicians’ obligations to their patients and to public health, then, point them toward prescribing shorter courses.

### Clinical Evidence in Favour of “Shorter is Better”

There is a growing movement in infectious disease medicine that holds that, with respect to antibiotic treatment durations for many uncomplicated common infections, “shorter is better” (Spellberg 2016). Proponents of the movement highlight that there is little reason behind specific traditional antibiotic treatment durations (Llewelyn et al. 2017). They also argue that instead of adhering to the traditional prescription lengths that are often arbitrarily based on medically irrelevant facts like the number of days in a week, physicians should prescribe shorter courses where this would be expected to have no negative effect on cure rates (Spellberg 2016). The claims are built around evidence, first, of arbitrariness in the history of setting prescription lengths (Radetsky 1990), leading to a negative claim that we should question current wisdom on prescription duration. It appears likely that the common prescription length of multiples of five or seven days is based on anything from the days in a week (Spellberg 2016), to Pythagoras’ sacred numbers 1, 3, 5, and 7 and their multiples, to the superstition that “The duration of antibiotic treatment is 5 or 7 days or multiples thereof” (Alp et al. 2005, 442). Indeed, work tracing the history and results of early penicillin trials against pneumococcal pneumonia clearly show evidence for equally effective treatment with shorter courses (Rice 2008), with early US guidelines released by the IDSA on the treatment of CAP reportedly indicating: “We are not aware of any controlled trials that have specifically addressed the questions of how long pneumonia should be treated.”(Rice 2008) That claim has been removed from versions after 2000, whilst the recommended treatment duration remained the same. Yet, there was no evidence at that point that longer courses reduced risk of resistance, relapse, or that relapse was associated with (net increase in) the emergence of resistance (Rice 2008).

There is also the positive claim that shorter is better, built on accumulating evidence from randomized trials showing that shorter lengths of antibiotic treatment are non-inferior to alternatives in terms of curing the patient (Chastre et al. 2001; Dawson-Hahn et al. 2017; Moh, Tan and Cooper 2025; Singh et al. 2000; Tansarli et al. 2016). Such evidence has been gathered, for example, for CAP, cellulitis, urinary tract infection, osteomyelitis, and many other bacterial infections (Spellberg n.d.) [Table 1].

For CAP in particular, there are 14 trials comparing short courses (3-5 days) to long courses (5-14 days) of antibiotics that all demonstrate equal cure rates for shorter courses (see Spellberg’s website (n.d.) for a longer version of the table below).

Alongside mounting evidence that shorter courses are non-inferior in terms of curing the patient, shorter courses are associated with fewer medication-related adverse events. One study shows that for every additional 10 days of antibiotic treatment, there is a 3% increased risk of adverse events including gastrointestinal, dermatologic, musculoskeletal, hematologic, hepatobiliary, renal, cardiac, and neurologic events (Tamma et al. 2017). The UK’s National Health Service guidelines make no mention of side-effects of amoxicillin for treating CAP, yet adverse events commonly include gastro-intestinal symptoms (e.g. diarrhea) and skin rashes (n.d.). Every dose of an antibiotic involves risk to a patient, with more doses meaning more potential harm, and fewer doses meaning less potential harm. Given there is no additional benefit in terms of curing the patient of CAP for antibiotic courses over 3-5 days, this means there is, in most cases, net harm expected to patients for antibiotic prescriptions longer than the minimum required for cure. The length of exposure to harms is reduced in short courses. It is therefore remarkable that so many physicians expose their patients to net harm from longer antibiotic courses in real world clinical practice.

So much for the evidence against longer courses and in favor of shorter courses as concerns the individual patient. Physicians also ought to consider the effects of shorter courses on risk of antibiotic resistance developing—with implications not only for the patient, but for global public health. Physicians and patients alike have been taught that patients must “finish the course” to avoid contributing to resistance—in other words, that under-treatment of the “target” bacteria causing the current episode of infection causes drug resistance. The implication is that the risk of this is greater than the risk of drug resistance emerging in other bacteria due to “off-target” effects of antibiotics on other bacteria in the body (which accumulate with every additional dose of antibiotics (Tedijanto et al. 2018)).

In fact, far more resistance can be expected to occur due to off-target effects of antibiotics on bacteria in the human microbiome, some of which have the potential to cause (resistant) disease in the patient in future, as well as to be transmitted to others (Jamrozik and Selgelid 2020; Llewelyn et al. 2017; Tedijanto et al. 2018). Whilst there is not yet enough evidence to make certain claims linking shorter antibiotic courses to reduced resistance in the microbiome for many common infections (Mo, Tan and Cooper 2025), we might logically expect a greater effect on the microbiome: the number of target bacteria causing any particular episode of infectious disease is minute compared to the number and variety of off-target bacteria present in the human body (Sender, Fuchs, and Milo 2016) [Figure 1]. The risk of off-target selection of resistance has been shown in some limited cases to be greater for longer antibiotic treatment duration (Lodise 2007). The longer that bacteria are exposed to selective pressure, the greater the opportunity for resistant organisms already present in a patient’s microbiome to replicate. There is also greater opportunity for the acquisition of new resistant organisms (or resistance genes) from others or the environment, which might occupy niches previously held by drug-sensitive organisms (Llewelyn et al. 2017). Insofar as people carrying resistant bacteria (whether symptomatic or not) pass them on to others and cause illness, ABR as a public health problem may well be exacerbated by these longer antibiotic prescription lengths.

On all measures of patient and public health, then, it is worse, overall, for physicians to prescribe antibiotic treatments any longer than the minimal length required to cure the patient. There is no ethical question faced by the physician: shorter antibiotic courses are better, all things considered.

### Ethical Importance of Clinical Evidence

To fulfil their ethical obligations, physicians must use their clinical reasoning and their understandings of available clinical evidence on the effectiveness of specific prescription decisions on cure rates, as well as adverse events and side-effects of treatment, and effects on public health more broadly.

Physicians’ duties toward both their specific patients and to protect public health more broadly arise from their professional guidelines and legal obligations, and the tradition of the profession. The American Medical Association (AMA)’s “social contract with humanity”, was adopted in 2001 and contains the oath that “We, the members of the world community of physicians, solemnly commit ourselves to… apply our knowledge and skills when needed”. (2001, art. 4) Similarly, the AMA’s code of medical ethics asserts professional obligations that the physician has toward their patient. Ethicists have argued that the duty of care, expressed in many of these ways, may include a duty to treat the patient (though the duty may be limited in certain circumstances such as during public health emergencies that put physicians in significant danger) (Malm et al. 2008). Professional standards of good medical practice set by the General Medical Council (GMC) of the UK require that physicians make “the care of patients their first concern” and protect and promote “the health of patients *and the public*.” (2024, italics added). It has also been argued that the virtuous physician considers both the patient and the community when making antibiotic prescribing decisions (Oakley 2020). Patient-centered medical virtues include medical courage and medical beneficence developed through practical wisdom, to determining whether a particular prospective prescription decision is in a patient’s best interests and to be willing to act on that judgement. Community-centered medical virtues include justice and readiness to serve the broader community (Oakley 2020). These virtues may lead a physician to make antibiotic-prescribing decisions that benefit the patient and community as far as possible, and that appropriately balance trade-offs where a patient’s interests conflict with what best protects public health.

### Refuting Objections

Given the evidence presented in the sections above, why do physicians continue to prescribe longer courses, and how might an objector to our argument defend such a decision? As a general argument, they might agree that there is now good evidence in favor of shorter antibiotic courses, but hold that there are several other factors that legitimately influence clinical decision-making. We have identified both legitimate and illegitimate influences, including practical considerations, systemic factors, and behavioral influences/cognitive biases. Whilst practical considerations and systemic factors may be outside a physician’s sphere of control to alter their prescribing practices, we hold that it is important that behavioral influences and cognitive biases in particular are addressed where possible by individual physicians. Longer-term and broader-scale change must be secured through policy and culture change, altering systems and structures that negatively influence or constrain physicians’ prescribing decisions.

#### Practical considerations

One practical consideration that might influence a physician’s prescribing decision is that it may be unlikely that a patient will return to a GP and book another appointment to see if they need a longer course of antibiotics. This might be particularly the case if the physician can either give an out-patient, say, a short three-day course of amoxicillin to treat pneumococcal CAP, or a longer five-day course. The physician may need to tell the patient that they must come back the following day for check-up if the three-day course is prescribed, whereas they need not rely on the patient to decide to attend another appointment if they simply prescribe a slightly longer five-day course. Rather than risk treatment failure in the event of further treatment being needed after a shorter course of antibiotics, physicians may feel it is “better to be safe than sorry” to prescribe a longer course. However, longer courses do not usually increase safety, neither in terms of cure nor in terms of other harms to patients as we have highlighted above. This view is therefore arguably misguided on the basis simply of safety, even without considering plausible changes to antibiotic prescription practices to allow patients to assess whether they need a repeat prescription of a short-course antibiotic and order one if needed without returning to the physician.

A second practical consideration may be the number of tablets in a typical antibiotic pack, which rarely aligns with the appropriate course length (McGuire, Smith and Del Mar 2015). This may influence physicians to simply prescribe one packet of antibiotics as a “course”, without reflecting adequately on whether the number of doses in a packet is appropriate for a specific patient’s treatment and aligns with clinical guidelines. However, such considerations do not obviate physicians’ duties to prescribe appropriate (in this case appropriately short) durations of therapy. In the longer term, packet size should arguably change to reflect evidence regarding shorter course durations.

A final practical consideration may be the lack of information readily available to physicians on the harms associated with antibiotic treatment. Guidance from bodies like IDSA and NICE in the UK on treating CAP fail to mention side-effects from antibiotics such as amoxicillin on the prescription directions page. In general, this information may be harder to find (Xiang, Heriot and Jamrozik 2023). Without this information, physicians cannot make adequately informed risk-benefit analyses of antibiotic treatment decisions. Guidelines need to be clearer, then, on the harms of antibiotic treatment. The lack of emphasis on harms arguably reflects wider systemic problems in medical education, research, and practice characterized by a focus on benefits and a relative neglect of harms (McDonnell Norms Group 2008; Stegenga 2016). This may result in physicians often under-estimating harms (and/or over-estimating benefits) and therefore overprescribing treatment.

#### Systemic factors

Systemic factors are those elements contributing to a physician’s prescribing decision that are influenced by external pressures, structures, and requirements. These may constrain a physician’s actions with regard to prescribing shorter courses of antibiotics, even if they acknowledge the evidence in favor of shorter-duration courses. This may lead to not an evidence gap, nor a cognitive bias, but rather an implementation gap in physicians’ abilities to adapt their prescribing decisions according to emerging evidence that ‘shorter is better’.

One issue is risk-avoidance, mediated not by the cognitive biases that we describe in the sub-section below, but due to structures in a healthcare system that focus on negative consequences of treatment decisions that are short-term and proximate to the patient, rather than adequately considering negative consequences of an alternative possible treatment decision that might be longer-term or more broadly-distributed across people or probabilistic. The investigation of safety incidents focuses in on physician errors and may take testimony from norms set within a medical hierarchy or norms of practice (NHS England 2015). These pressures may lead physicians to prefer to avoid risks of under-treating the target infection (therefore prescribing a longer-than-guideline-minimum course of antibiotics).

Another systemic factor is frequent under-staffing in healthcare systems. Where there is a misalignment between the time a physician needs to thoroughly assess a patient’s symptoms and prognosis, and the time they are allocated to see a patient, there is greater risk of physicians being constrained to simply relying on fast decision-making processes instead of detailed assessment (Sanford et al. 2022). Workflows may also be altered in understaffed systems, with unanticipated changes in workflow influencing antibiotic prescribing decisions for surgeons, particularly in systems where timeliness of antibiotic administration is not prioritized (Charani et al. 2011). Faster decision-making may tend to refer more to ‘prescription etiquette’ established by other physicians in that care setting, to ‘better safe than sorry’ attitudes, and to clinical guidelines. There is a risk of physicians being led to prescribe longer courses than clinical guidelines recommend as minimum, when these fast decision-making influences are combined, particularly for more junior doctors who do the majority of antibiotic prescribing in many settings, and who may tend to comply more with senior physicians’ prescribing tendencies (Papoutsi et al. 2017).

Some of the considerations we have outlined thus far are warranted as influences on physicians’ prescribing decisions, due to ways in which the current set-up of the healthcare system, particularly in the US, demands that physicians consider medico-legal consequences of their actions and risks to reputation. In addition, physicians may have little time to keep up to date with emerging evidence given under-resourcing. However, we maintain that in physicians’ decision-making, ideally, there is no place for such influences that lie beyond clinical relevance. What’s more, they often go hand-in-hand with two important issues we discuss next: cognitive biases and underestimating the potential harms of antibiotics, which seems to underly the phrasing of the ethical argument expressed above.

#### Behavioral influences and cognitive biases

Behavioral influences and cognitive biases also feature in clinical decision-making (Janssen et al 2022). In fact, they may be more powerful than other considerations: studies show that duration of antibiotic treatment of CAP, for instance, is impacted neither by disease severity at time of hospital admission, nor by clinical response to therapy (Aliberti et al. 2010). What does influence duration of antibiotic treatment may be factors such as deference to more senior physicians (Livorsi et al. 2015). One study highlights a tendency for “eminence over evidence” in the formation of behavioral norms among trainee doctors. This often goes alongside a perception of omission as a sin much graver than commission (McDonnell Norms Group 2008). As stated by one interviewee in a study by Pandolfo et al. of behavioral influences on prescription:

I think they [intensive care doctors] would be always being erring on the side of caution, you wouldn’t even stop [antibiotics] if there is any suspicion of risk [of bacterial infection]. (2022, 202, P40).

Such thinking is misguided, as we explain below. It may, furthermore, be based in part on fear of medico-legal consequences, as noted by hospital consultants in Pandolfo et al.’s study:

One of the questions that I was grilled on in the Coroner’s court last Monday was ‘Why are we stopping antibiotics? If he had such a bad infection, why did we stop the antibiotics?’ (2022, 203, P28).

Even if there was good reason to think that stopping antibiotics “early” would have no effect on the chance of patient recovery, physicians might want to avoid being cross-examined on the decision to stop antibiotics in court.

Finally, physicians may feel there are ethical arguments in favor of longer treatment lengths. Physicians have a duty of care toward their patients, and physicians might consider that specific patient factors warrant longer treatment on the basis of a duty of care for that particular patient and their greater need for antibiotics. A participant in Pandolfo *et al*.’s study reported that

I think a four days’ course of antibiotics is going to lead to resistance if anything because he’s not completed a full course […] so I think a seven-day minimum [course] would be appropriate. (2022, 203, P22).

However, for ordinary episodes of CAP this will rarely if ever be the case given that multiple studies of CAP have showed no statistically significant benefit of longer courses. Professional ethical codes require physicians to, for example, “make the care of your patient your first concern” and “protect and promote the health of patients and the public” (General Medical Council n.d., 1). It is part of the knowledge, skills, and ethical practice required of a physician give their patients the treatment option that has the best balance of benefits over harms for that patient, all things considered (Haynes 1999). Given that every dose of antibiotic carries risk, patients face net expected harm for treatment beyond the minimum necessary for cure. Likewise, excessively long antibiotic courses may produce risks to public health from contributing to the spread of ABR in addition to net harm for patients.

One cognitive bias might be for physicians to underestimate the harms of overprescribing antibiotics. Underestimating harms can result from ignorance, or from weighing benefits and harms differently. On the first point, physicians may simply not be aware of evidence of the effectiveness of shorter courses (Pandolfo et al. 2022). In other cases, physicians may be subject to the practical considerations regarding their ability to anticipate harms due to the limitations of prescription guidelines that we described above. Physicians might nevertheless be considered at least partially responsible for attempting to correct their own biases, such as risk avoidance. Risk avoidance is a common cognitive bias where harms are considered to weigh more than benefits, and it is not a bias to which we might (at least initially) expect physicians to be immune. Risk avoidance is associated with increased defined daily doses in a study on antibiotic prescription determinants in Europe (Harbath and Monnet 2008). It may be that the salience of one risk—rare poor clinical outcomes associated with CAP, perhaps perceived to be associated with shorter treatment—is greater than the salience of another risk—poor clinical outcomes due to adverse events associated with the antibiotics and harms due to the development of (more) resistant bacteria in the patient’s microbiome. This discrepancy in the consideration given to infection-derived harms compared to antibiotic-associated harms has been demonstrated in qualitative studies on prescribers (Livorsi et al. 2015). The same bias might apply to perceived risks to public health, wherein the risk of resistance developing in the disease-causing pathogen is perceived as more salient than the risk of resistance developing in other bacteria in the patient’s microbiome. This combination of salience effects and risk avoidance might alter how physicians weigh risks and benefits of longer antibiotic treatment courses. On the other hand, it is important to note the importance of physicians ascribing appropriate weight to low-probability but high-severity outcomes; in some clinical cases where physicians are aware of risks of very severe health implications for patients associated with failure to treat the target infection, it would not be a cognitive bias for them to ascribe *appropriate* weight to this consideration and be guided by it in their clinical decision-making. This may limit the implications of our argument in favor of physicians prescribing shorter antibiotic courses than they typically do, and anything shorter than treatment guidelines currently recommend, for complicated or high-risk infections (da Silva Perez Bezerra et al. 2020). However, even complex infectious disease syndromes may be appropriate targets for research, as we discuss below. In contrast, given current evidence for many infections that under-treatment does not pose a greater risk of harm than over-treatment to either the patient (considering both probability of cure and adverse events) or to public health (considering risk of resistance), physicians should aim to control these cognitive biases and rely instead, on the evidence showing that shorter is better when it comes to antibiotic prescription lengths. Indeed, physicians have an ethical obligation to stay updated on current medical evidence relevant to their sub-field, to fulfil requirements of the biomedical ethical principles of beneficence and non-maleficence when treating their patients (Bhugra 2024).

An important final caveat is that clinical judgement is essential as a part of the decision-making process where physicians choose between various options for prescribing antibiotic course durations. Alongside relying on the evidence base and clinical guidelines, physicians should exercise their medical expertise and use their advantage in being able to assess the individual patient directly. It is for this reason that it is important that, as the shorter is better movement advocates, there is still a *range* of antibiotic course durations open for physicians to prescribe. A single, pre-set duration would not allow room for clinical judgement, and might produce worse patient outcomes. However, the fact that average prescription lengths consistently exceed guidelines suggests that clinical judgements are indeed biased toward overtreatment—the norms of clinical judgement should therefore be updated in line with current evidence while still leaving room for *rare* situations in which longer courses may be justified.

Following these objections and our caveat here, we continue to support the argument that physicians have an ethical obligation to prescribe shorter antibiotic course durations than they tend to do, insofar as this accords with the evidence base and clinical guidelines, and in cases of uncomplicated common infections like the pneumococcal CAP case we present. This is because shorter treatment: (i) produces equivalent clinical outcomes for the patient in terms of curing them of their disease because the target infection is eliminated within the shorter treatment time, (ii) reduces individual risk of developing or acquiring resistance (compared with longer treatments) because of shorter exposure of the microbiome to selective pressures of antibiotics, and (iii) produces better public health outcomes through less contribution to the population prevalence of ABR because of the reduced risk of resistance emerging in the individual’s microbiome. Furthermore, it is clear by assessing the effects of longer treatment that shorter treatment is ethically preferable. Longer treatment: (iv) produces worse clinical outcomes for the patient in terms of side-effects of antibiotic use which cause discomfort or harm, (v) exposes patients to a higher risk of developing resistant bacteria through increased microbiome exposure to antibiotics, and (vi) produces worse public health outcomes through greater risk of contribution to ABR. We present the pros and cons of shorter treatment compared to longer treatment in [Figure 2]. In the case of pneumonia, for example, physicians should offer shorter courses of at most 5 days of antibiotic treatment for those suffering mild to moderate CAP.

### Ethics of Clinical Trials of Shorter Antibiotic Treatments

At this point, we have argued that the physician from our original example faces no ethical question relating to her treatment of CAP concerning whether she should offer a shorter or longer course of antibiotics. She should prescribe at least as short a course as guidelines recommend. In this section, we explore the ethical case in favor of (further) clinical research testing (even) shorter courses of antibiotic treatment than those recommended by current guidelines; this may lead to a future where under certain circumstances, it is, arguably, ethically obligatory for physicians to prescribe antibiotics of shorter duration than guidelines recommend. One potential objection to clinical trials testing shorter courses (including those that have already been conducted to date) is that it would be unethical to trial a comparator arm with a shorter antibiotic course, given widespread practice and/or evidence in favor of the (longer) standard of care and a lack of incidental evidence in favor of shorter treatment durations (Spellberg 2016). However, even if many prescribing physicians hold such views, this does not undermine the ethical case for treatment-shortening trials. There are several reasons for this. First, such intuitions regarding risks of reduced cure rates due to shorter courses have been shown to be false in treatment-shortening trials to date, as discussed above. Second, there is a diversity of expert views about appropriate treatment durations, as discussed further in the next sub-section on equipoise. Third, because outcomes other than cure also matter, including the risks of antibiotic-related adverse drug reactions and the risks of developing (more) resistant bacteria in patients’ microbiomes. There are therefore multiple reasons why appropriately designed treatment-shortening trials are ethically acceptable – there may even be a strong ethical case in favor of such trials, given the individual and population-level harms associated with overtreatment. Further, since the shortest duration of antibiotic treatment with equal effectiveness to longer courses is often unknown, it may be ethical to randomize participants to very short courses—even if this entails the possibility of reduced cure rates, provided that patients are monitored for these outcomes in order to mitigate relevant risks (Walker et al. 2025). However, if standard practice for certain infections can be reduced to relatively short durations based on current evidence (e.g., 3 days for CAP) then trials of shorter courses might not make a large impact on practice at the individual patient level (e.g., because changing to a 2 day course would represent a reduction of only 1 day).

In general, there will be a stronger ethical case for trials of shorter treatment durations where (i) current treatment durations are longer, (ii) evidence supporting current treatment durations is weak, (iii) the clinical syndrome is common, (iv) antibiotics are associated with significant adverse effects and/or selection for resistance, and/or (v) patients are treated with antibiotics often and/or more likely to be harmed by the carriage of resistant bacteria (e.g., immunosuppressed patients or those requiring frequent admissions to hospital). Many current clinical syndromes meet one or more of these criteria, for example diabetic foot infections including osteomyelitis involve long treatment durations (of weeks or months) in patients who suffer harm from recurrent treatment-resistant bacteria (Spellberg, n.d.).

#### Equipoise

When we interrogate potential objections to clinical trials for shorter-still courses, one relevant concept for discussion is equipoise: a state of collective expert uncertainty or disagreement regarding the comparison between two treatment options (Freedman 1987; Fried 2016). Equipoise is widely considered to be a requirement for the ethical conduct of clinical trials, because the absence of equipoise suggests that researchers would fail in their duty to consider the welfare of research participants by giving some participants a less effective treatment than the standard of care. However, we ask why there might be a perceived lack of equipoise in this area that could justify research. While there may be widespread dogmatic beliefs among physicians regarding the superiority of longer courses of antibiotics, these are not based on high-quality evidence. Where a consensus view based on minimal evidence is dogmatically held by most experts in a field, or most senior experts, this can foreclose or delay opportunities for innovative research ideas (e.g., to trial shorter-still antibiotics courses) (Barosa, Jamrozik and Prasad 2024). Further, the ethical acceptability of research (and concepts of equipoise) should not depend on beliefs based on minimal evidence, especially where there are good reasons to think that those beliefs may be mistaken. Finally, such beliefs, although widespread, are not universal, and there is arguably significant scientific uncertainty as well as disagreement among experts regarding optimal antibiotic course durations.

For many infections, longer antibiotic courses have already been shown inferior to shorter courses in terms of overall patient outcomes, so there might be justification for trialing very short courses. If evidence from clinical trials of shorter *vs* shorter-still antibiotic treatments shows non-inferior cure rates for shorter-still courses, then these new even shorter courses should become the standard of care. Given equivalent cure rates, reduced chances of resistance developing and reduced risk of adverse events and given physicians’ duty of care to their patients and duty to protect public health, physicians would arguably be ethically obligated to prescribe shorter-still courses of antibiotics.

### Implications For Other Diseases

The arguments we make in this paper may also apply to other infections where antibiotic treatment is indicated, *viz*. arguments regarding (i) physicians’ duties to prescribe shorter antibiotic courses given evidence that “shorter is better”, (ii) the ethical importance of conducting clinical trials for shorter-still treatment courses, and (iii) the ethical implications for physicians if or when even shorter courses are shown in future trials to have equivalent cure rates. The implications may extend beyond CAP to many other common uncomplicated bacterial infections in healthy individuals such as urinary tract infections, skin infections, and gastro-intestinal infections (See [Table 1] for examples).

The implications of this work may also apply to some more invasive infections; for example, recent research has even shown that bloodstream infections can be successfully treated with much shorter courses of antibiotics than have been previously used (e.g., 7 days instead of 14 days) (Yahav et al. 2019). However, complicated infections such as those involving abscess formation or infection of foreign bodies such as joint prostheses, often require surgical intervention (sometimes referred to as “source control”) in addition to antibiotics. Yet, insofar as antibiotics are used once source control is achieved, it may often be ethically desirable to trial shorter courses if these have not previously been shown to be less effective than longer courses, for similar reasons as those we have advanced above.

We note that these arguments apply even to some types of infections such as tuberculosis (TB) where courses of several months’ duration are common and resistance is a major issue that is often presumed to be due to failure to complete antibiotic courses (frequently due to a lack of reliable access to longer-term antibiotics in low-income countries). For at least some diagnoses of TB, including some MDR TB strains, trials of shorter courses (6 months) of standard therapies have recently been shown to be similar in terms of cure to longer courses (e.g., >9 months (Esmail et al. 2022)). However, for diseases like TB where resistance is a major concern, and treatment duration as well as post-treatment follow-up to assess for cure may be long, trials of shorter courses may need more carefully designed and may need longer-term research funding investment (Connolly, Edelstein and Ramakrishnan 2007; Walker et al. 2025).

These arguments likely apply not only to infections in otherwise-healthy people but also to many infections in immunocompromised individuals. For example, it has been shown that long durations of antibiotics are rarely required for uncomplicated febrile neutropenia (a common syndrome in patients undergoing chemotherapy that is often presumed to be due to bacterial infection in an immunocompromised host) (Imlay et al. 2023). Although immunocompromised patients are at high risk of serious bacterial infections (and thus many physicians may be uncomfortable in prescribing or trialing shorter courses), they are also at high risk of being over-treated with antibiotics and suffering antibiotic side effects as well as subsequent infections due to resistant pathogens arising due to off-target effects of antibiotics. Thus, it is in the interest of immunocompromised patients that more trials are done to determine the shortest course of antibiotics that can cure common bacterial infections in these groups. Of course, there may remain many cases where specific factors about the patient or the bacteria causing a given infection may support the use of long courses of antibiotics. However, these will represent a minority of cases, and our arguments for shorter courses likely hold true for a wide range of different infections, building on clinical evidence of the effectiveness of shorter treatment lengths (Yahav et al. 2019).

Overall, this work has significant implications for the optimal use of antibiotics worldwide. Trends in the UK and the US, where 70-80% of antibiotic prescriptions for cases such as respiratory tract indications exceed guidelines in antibiotic course duration (Yi et al 2018; Pouwels et al. 2019) are likely to be similar in many other countries. Further, guideline course length sometimes exceeds the best available evidence on shorter courses, suggesting that even prescribing according to guidelines may result in overtreatment. Given such widespread overuse, significant global public health gains could be made through changes to prescription behaviors with no resultant expected harm to patients from reduced cure rates (and potential benefits for patients in terms of lower rates of adverse drug reactions and carriage of resistant bacteria in their microbiomes).

Policies to improve education among physicians, patients, and the public could do much to reverse the harms of the “finish the course” mantra taught in previous decades (Llewelyn et al. 2017), and, beyond education, more interventionist policies may be appropriate in some settings, to encourage prescribing physicians to stick to shorter prescriptions where indicated by clinical guidelines and evidence. This behavior change is not merely preferable—it is arguably urgent in the face of the rising threat of antimicrobial resistance.

## Conclusion

We have argued that physicians’ attitudes toward antibiotic prescription need to change, because their current decisions are causing harms to patients and public health. Physicians concerned with their patients’ best interests and with public health ought to be more concerned about over-treatment and less concerned about under-treatment with antibiotics. Clinical guidelines, medical evidence, and ethical considerations clearly favor shorter courses. Further clinical trials should be conducted to test the effectiveness of even shorter antibiotic courses to determine the shortest possible effective course for common infections. These changes are needed urgently to reduce harms to individual patients, and to protect public health from likely further unnecessary contributions to ABR.

## Figures and Tables

**Figure:**
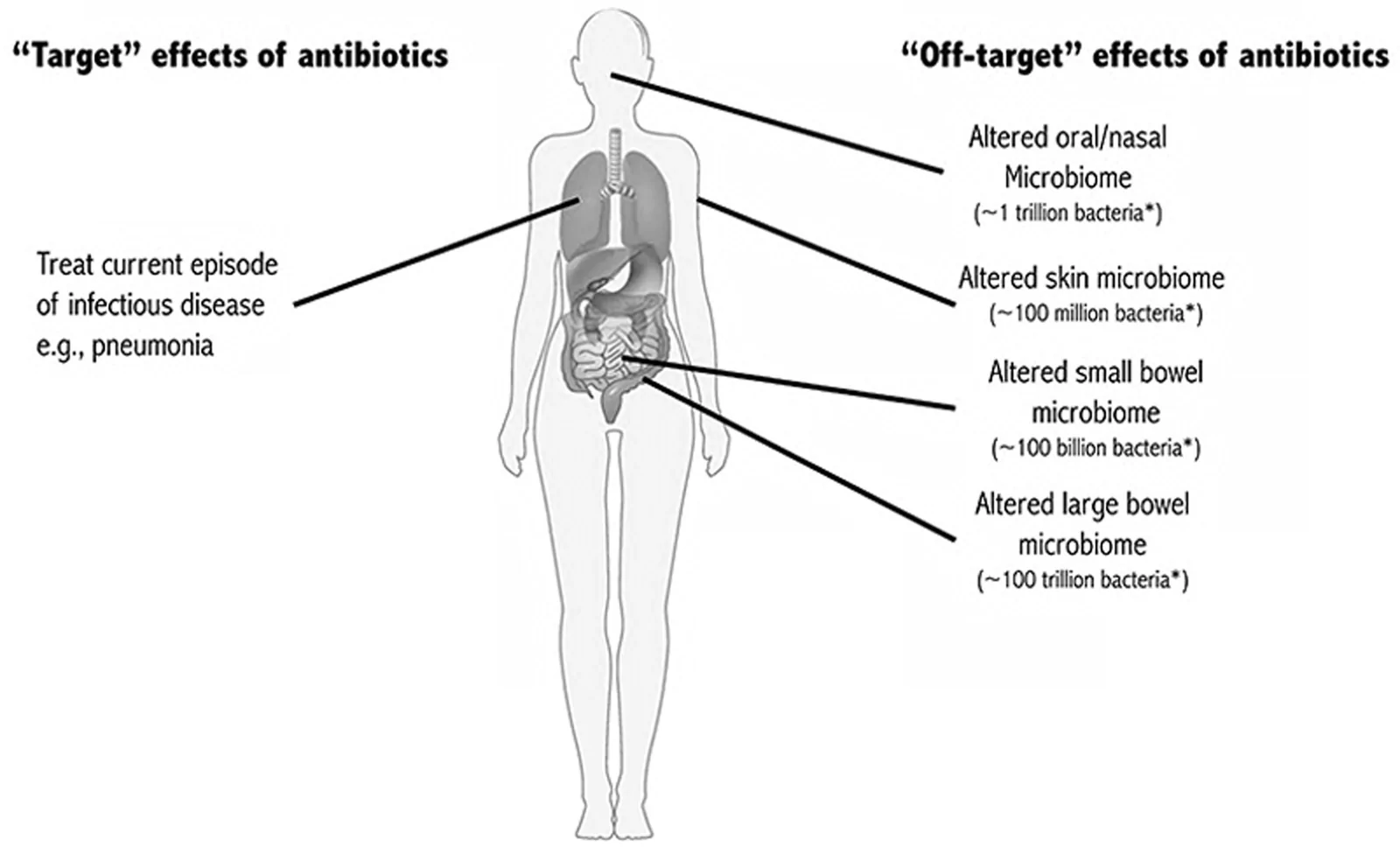


**Figure:**